# Long-term outcomes of magnetic sphincter augmentation in sleeve gastrectomy and Roux-en-Y gastric bypass patients: a comprehensive analysis

**DOI:** 10.1007/s00464-024-11059-4

**Published:** 2024-07-19

**Authors:** Mina A. Ibrahim, Daniel P. Mowoh, Mai Al Khadem, Mujjahid Abbas, Leena Khaitan

**Affiliations:** Department of Surgery, Cleveland Medical Center, Case Western Reserve University School of Medicine, University Hospitals, 11100 Euclid Avenue, Cleveland, OH 44121 USA

**Keywords:** Bariatric, Magnetic sphincter augmentation, Revisional surgery, Anti-reflux surgery

## Abstract

**Introduction:**

Management of gastroesophageal reflux disease after bariatric procedures can be challenging. There are very few long-term studies in this arena. This study aims to evaluate the long-term outcomes of the magnetic sphincter augmentation (MSA) reflux management system in a cohort of bariatric patients who had previously undergone sleeve gastrectomy and Roux-en-Y gastric bypass, with a focus on assessing gastroesophageal reflux disease (GERD) scores, medication use, and patient-reported symptoms.

**Methods:**

We conducted a retrospective chart review of 16 consecutive bariatric patients who received MSA implants following sleeve gastrectomy (*n* = 14) or gastric bypass (*n* = 2) surgeries. Data were collected regarding BMI, GERD quality of life assessments (GERD-HRQL), reflux symptoms, and use of PPIs in the sleeve/RGB patients through an extended period with a mean follow-up of 48 months.

**Results:**

Patients were followed up for a range of .5–84 months. Preoperative assessments included upper gastrointestinal imaging (UGI), high-resolution manometry, Bravo pH studies, and esophagogastroduodenoscopy (EGD). Three patients exhibited reflux on UGI, and 13/13 patients had positive Bravo studies preoperatively. Sixteen patients had a lower esophageal sphincter (LES) pressure under 18 mmHg, and eight patients had biopsy-proven esophagitis. Long-term outcomes are as follows. Daily PPI use fell from 88 to 25% at greater than three years. GERD-HRQL scores fell from 50.6 at baseline (range 27–70) and normalized at long-term follow-up. GERD symptom of regurgitation completely resolved. At long term, two patients had dysphagia and two patients had ongoing reflux. No adverse events were noted.

**Conclusion:**

This is the first long-term outcomes study of magnetic sphincter augmentation placement after bariatric surgery. Our study showed the majority of patients had long-term improvement in GERD-HRQL scores and resolution/ relief of their reflux symptoms, with decreased use of PPIs. MSA is a safe, effective and durable management tool for reflux after bariatric surgery in carefully selected patients.

In the USA roughly one out of every five individuals carries a diagnosis of gastroesophageal reflux disease (GERD) [1]. The prevalence of GERD is greater among the obese population with a significant correlation between body mass index greater than thirty-five and increased reflux symptoms. Since bariatric surgery has gained significant popularity and remains the mainstay for the durable management of class III obesity, there is an increased focus on the management of postoperative reflux in these patients [2, 3]. The two most performed operations for weight loss in the USA are a sleeve gastrectomy (SG) and Roux-n-Y gastric bypass (Roux-en-Y gastric bypass). Although there has been significant advancement in diagnosing GERD following bariatric surgery, there continues to be ongoing debate regarding the management strategies for these patients [4].

It is also well known that patients who undergo sleeve gastrectomy have an increased incidence of new onset reflux as high as 20–35% [2, 5]. Traditionally these patients with refractory GERD after sleeve gastrectomy are converted to a bypass [6]. Interestingly, there are also patients who undergo a primary Roux-en-Y gastric bypass who develop GERD symptoms refractory to medications and managing this is also challenging [7]. Techniques suggested to treat this condition, such as fundoplication with the gastric remnant, gastropexy to the arcuate ligament, and teres ligament repair, have all been proposed [7]. Since the gastric bypass itself is considered an anti-reflux procedure, one might wonder about these patients having GERD. The pouch still has acid-producing cells. In addition, in unaltered gastric anatomy, two of the primary causes of reflux disease are attributed to hiatal hernias and dysfunction of the lower esophageal sphincter. These mechanisms are possible in the post-bypass patient.

Magnetic sphincter augmentation (MSA) reflux management system has grown in popularity since its FDA approval in 2012 as an anti-reflux modality. This device works to augment the pressure within the lower esophageal sphincter and provides a better barrier to reflux of gastric contents into the esophagus. However, placement of MSA device in post-bariatric surgery patients was considered an off-label use until recently [8]. The RELIEF study demonstrated safety and efficacy of magnetic sphincter augmentation in the patient with persistent GERD following sleeve gastrectomy [9]. Now placement of MSA after gastric sleeve is considered on label use.

There are currently no long-term studies assessing the safety and efficacy of LES augmentation with MSA in the patient following bariatric surgery. Its use in the bypass patient remains off-label and very few reports exist in this population. The goal of this paper is to evaluate the long-term outcomes of the MSA reflux management system in a cohort of patients within a single institution who previously underwent a sleeve gastrectomy or Roux-en-Y gastric bypass and suffer from persistent GERD after bariatric surgery. Our primary outcome is long-term patient-reported GERD-HRQL scores after MSA placement. Secondary outcomes include patient-reported symptoms, use of proton pump inhibitors, and device tolerance.

## Methods

**Fig. 1:**
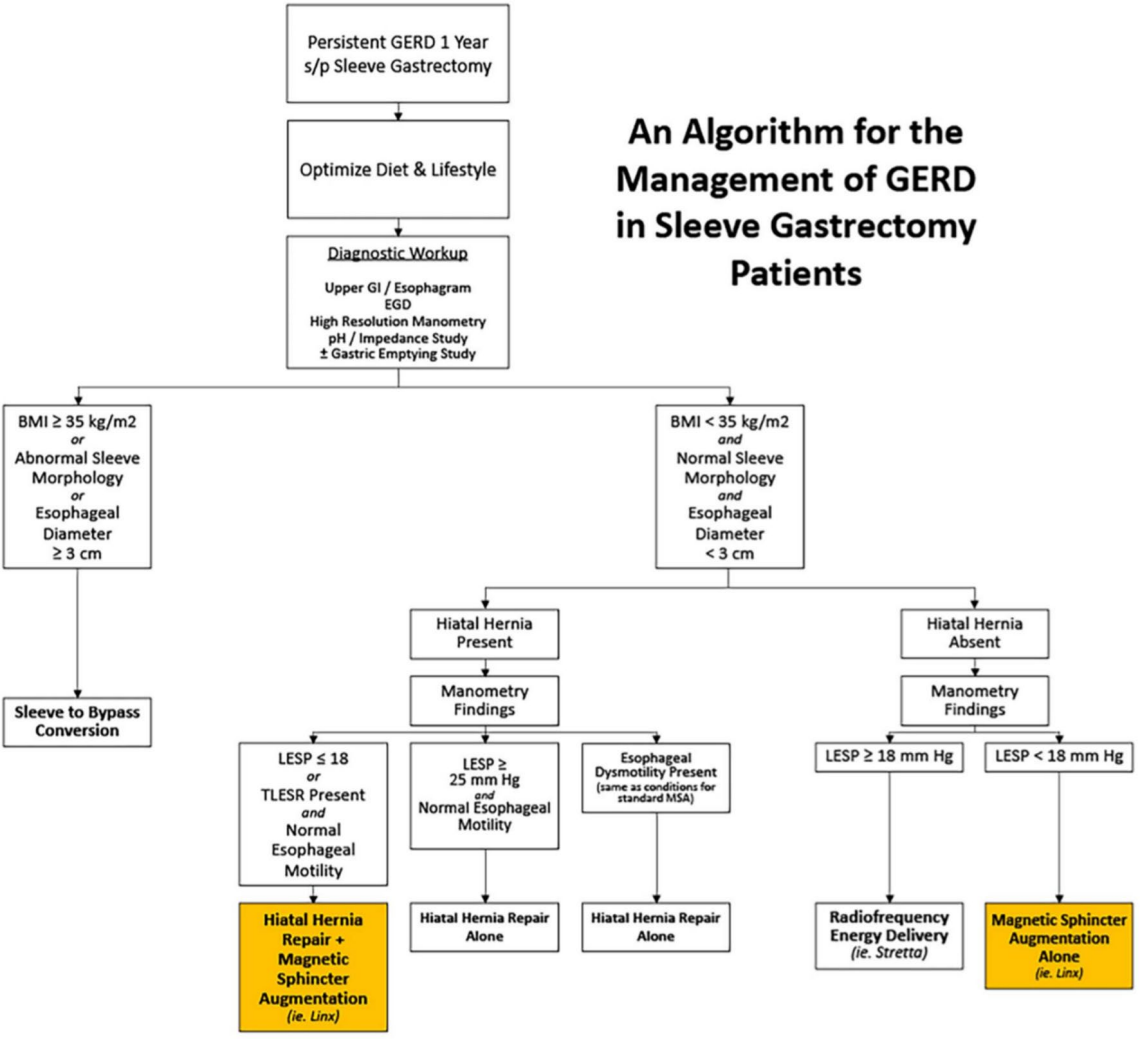
Algorithm for MSA selection in post-bariatric surgical patients with GERD [10]

Using a retrospective chart review within our system at University Hospitals of Cleveland, patients with a prior sleeve gastrectomy or Roux-en-Y gastric bypass with severe reflux were included if they underwent the placement of MSA implant from the years 2016 to 2023. From this database, 16 patients were selected, 13 of which had a SG, 2 had Roux-en-Y gastric bypass, and 1 patient had conversion of sleeve to bypass with concurrent placement of MSA. Patients underwent a thorough workup prior to placement of the MSA device. Preoperative assessments included upper gastrointestinal imaging (UGI), manometry, Bravo pH studies, and esophagogastroduodenoscopy (EGD). GERD-HRQL scores were used to assess severity of symptoms before and after surgery. Patients were followed at the following postoperative timepoints at 2 weeks, 1 month, 1 year, and at 3 years or greater. Short-term duration was defined as ≤ 1 year and long term was defined as ≥ 3 years. Mean follow-up time was 48 months ± 15 months. Demographics elements collected include age, gender, BMI, and procedure. Results of all studies were collated. Proton pump inhibitor (PPI) use, subsequent surgeries, and the number of beads within the device were all collected. Patient selection for placement of MSA was based on the following algorithm (Fig. 1). Evaluative studies were used to guide approach to management of reflux after bariatric procedures. Briefly, patients who had normal sleeve morphology, LES pressure under 18, normal motility, and BMI under 35 were considered candidates for MSA placement.

## Results

Patients were followed up for an extended range from 0.5 to 84 months. All of the patients were female. Preoperative age ranged from 36 to 64 years of age with a mean of 51 years of age. Preoperative BMIs ranged from 22 to 41 with an average BMI of 33. All the patients underwent EGDs (16/16, 13 pts had bravo studies, 16 patients had manometry, 13 patients had UGIs preoperatively). Three patients exhibited reflux on UGI. Using demeester scores greater > 14.7 and acid exposure time > 6% to indicate positive studies pH-Bravo studies. Low basal LES pressures were defined as LESP < 18 mmHg. Sixteen patients had a hypotensive LES at rest with a median value of 5, SD ± 5. IQR = 10.85 with a median value of 5.

Endoscopic findings were as follows. Esophagitis was noted in eight patients during preoperative upper endoscopy. Four patients had Grade A esophagitis and 4 patients had Grade B esophagitis when endoscopy was performed on PPI’s. One patient had nondysplastic Barrett’s esophagus. Six of sixteen (38%) patients had a hiatal hernia repair at the time of MSA placement. From the 6 patients with hiatal hernias the range was 2–5 cm (3 patients had 2-cm hiatal hernias, 1 patient had 3 cm, 2 with 4 cm, and 1 patient with 5 cm) which required repair. Fourteen patients were on daily PPIs prior to MSA device placement. GERD quality of life assessment scores at baseline varied from 27 to 70. GERD symptoms included typical symptoms that included dysphagia, reflux, and regurgitation, with reflux being the most common. Three patients had atypical symptoms with two patients having choking symptoms and one patient had foaming at the mouth. The most common symptoms were patient-reported reflux (16/16), followed by regurgitation (8/16) (Table 1).

**Table 1:**
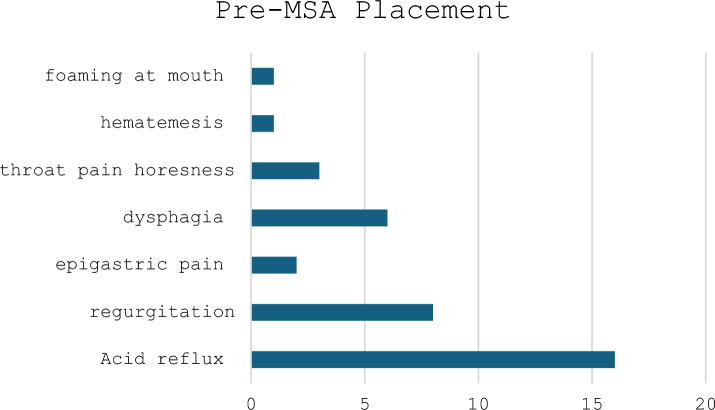
Pre-MSA patient-reported symptoms

From the patients’ population, 14 had a prior sleeve gastrectomy and 2 had a prior Roux-en-Y gastric bypass. One patient was converted from a sleeve to Roux-en-Y gastric bypass at the time of MSA implantation as the patient’s BMI was 41. Of the patients that were able to be followed long term, 8 patients were able to be followed greater than 3 years. 16/16 were followed at 2-week post-op, 15/16 at 1-month follow-up, and 12/16 were followed at 1 year.

Of the 8 patients (5 sleeve, 2 bypass, and one a conversion from sleeve to bypass patients) followed at the > 3-year mark, there was significant improvement in GERD-HRQL scores and symptoms (Table 2). Table 2 describes the trend of GERD-HRQL, patient-reported symptoms, and BMI prior to MSA placement postoperatively. Regurgitation resolved at long-term follow-up. Thirteen patients continued daily PPI use on the initial postoperative visit as this was part of the postoperative protocol. At greater than 3 years, only 25% (2 sleeve patients) remained on a daily PPI for management of GERD. The MSA devices used had a mean number of 16 beads with a range of (14–17), SD ± 1.

**Table 2:**
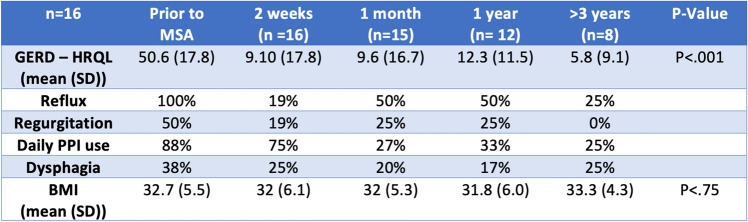
Trend of patient symptoms postoperatively

*(*n* number of patients, *SD* standard deviation)

**Fig. 2:**
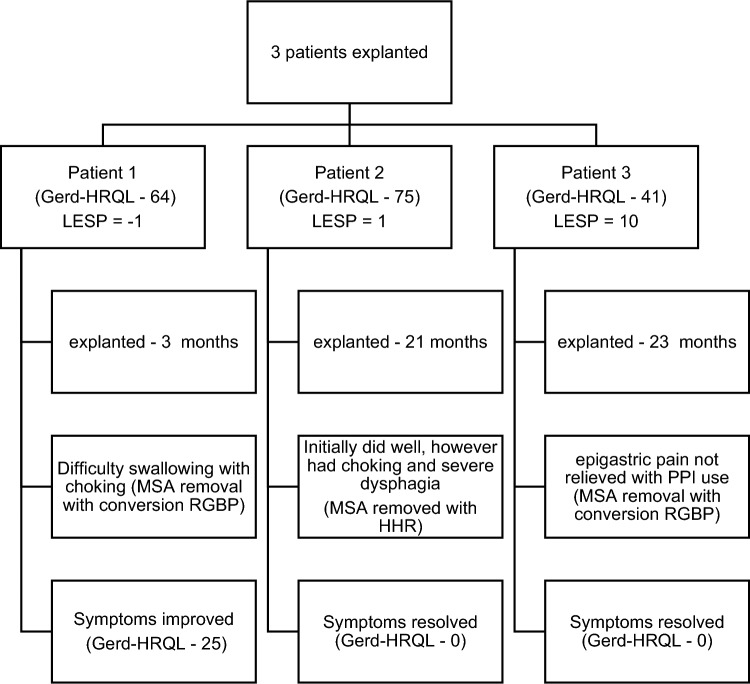
Explanted patients’ analysis

Three patients required their MSA device to be removed. Explantation rate was 19% (3/16). Of these patients, 2 had severe dysphagia (21 mo, 3 mo) requiring explantation. The indication for removal for one patient was severe epigastric pain (23 mo) (Fig. 2). Figure 2 outlines explantation patient breakdowns. 2/3 explanted patients had a very low LESP at the time of implantation (−1 and 1). 2/3 explanted patients were converted to bypasses. There was no device erosion or migration.

## Discussion

This is the first long-term outcomes study (> 3 yrs) of magnetic sphincter augmentation placement after bariatric surgery. Although there are limited studies in the use and success of MSA implantation in bariatric patients, none have shown its benefit beyond the 3-year mark. Our study showed that GERD-HRQL scores normalized, regurgitation resolved in all our patients, and daily PPI used decreased when evaluated long term. This suggests that the MSA is a durable option for post-bariatric surgery patients suffering from GERD in a carefully selected group.

Various studies have shown that the MSA is both efficacious with minimal adverse events [9]. Riva et al. performed a meta-analysis that showed significant improvement in GERD-HRQL scores with a median follow-up of 13 months (3.5) [11]. Broderick et al. demonstrated effectiveness of MSA following bariatric procedures as well. [12] Similarly, our study showed significant improvement in GERD-HRQL to a normalized value, both in a short-term and long-term course. Notable in both reviews was the improvement in HRQL and that daily PPI use significantly decreased. In fact in our study regurgitation also completely resolved. In the RELIEF study, resolution of regurgitation was observed and this was the symptom the patients related to their satisfaction most [9]. When it comes to management of reflux, symptom relief and quality of life are patient-reported measures that are an important measure of success. This was highlighted by the RELIEF study in which normalization of pH was not achieved; however, patient satisfaction remained high. One of the limitations of our study is the lack of objective measures of acid exposure in the long term; however, none of these patients had complications of reflux with strictures or other issues. They all demonstrated an improvement in quality of life and patient-reported measures with the intervention. This study further highlights that the effect of MSA is durable in the long term. Careful patient selection is noted as a big part of seeing this success in this population.

Traditionally patients with severe reflux post-bariatric surgery pose a significant challenge and the most common management strategy is conversion of sleeve gastrectomy to RYGB. Although this strategy is effective in many patients, there are those in whom it is not. Why is that? When traditional GERD management is discussed, the focus is on the hiatus in the diaphragm and also the lower esophageal sphincter. When a sleeve is converted to a gastric bypass for the management of reflux, neither of those etiologies of GERD is addressed. Then why does it work? Many surmise the effects are twofold. One is that the bypass may lead to further weight loss and this can help the symptoms of reflux. Secondly, the gastric bypass construction is a low pressure system. The sleeve is a high pressure system [12]. The conversion of the sleeve to bypass likely decompresses the pressure within the sleeve and therefore, the LES is less likely to be pushed open and therefore, the patient has less reflux. In one study looking at pressures within the sleeve, a distended sleeve was noted to have pressures as high as 43 mmHg. Knowing that LES pressure ranges from 10 to 45 mmHg, it is likely that LES can be pushed open easily by the sleeve anatomy causing reflux of gastric contents into the esophagus. None of the explanted patients were among the bypass patients in this series.

Patients that undergo revisional surgery with conversion of sleeve to bypass note that up to 22% risk of patients continue to experience GERD [13]. In part this may be because the LES dysfunction is not addressed and this correction maybe a major component in GERD symptom relief. It is also important that any hiatal hernias be repaired when managing GERD after sleeve gastrectomy. There is a growing body of literature supporting that the hiatal repair contributes greatly to the management of GERD, more so than the sphincter [14]. Hiatal hernias require surgical intervention and need to be repaired in all patients. In this series, 38% of patients had a concurrent hiatal hernia repair. Any noticeable hiatal hernia was fixed at the time of surgery and although only 6/16 had their hernia repaired at the time of surgery, a crural dissection was performed on all the patients to expose the GE junction and LES for proper device placement. When a hiatal hernia repair was performed it composed of a circumferential crural dissection, reduction of hernia sac, adequate intra-abdominal esophageal length, and mediastinal dissection [15, 16]. It is well known that LES augmentation with the MSA device as a stand-alone treatment in the absence of a hiatal hernia has a benefit. This was considered an important aspect of managing patients’ GERD.

Augmentation of the lower esophageal sphincter is more challenging after sleeve gastrectomy. Multiple approaches are discussed in the literature, including falciform ligament repair. There is no fundus that can be used for plication. However, if patients have some retained fundus, authors have described performing a partial plication with that [17]. Endoscopic management of the LES is very poorly researched in the post-sleeve population. This leaves magnetic sphincter augmentation as a very viable option. Prior to the RELIEF, MSA for post-bariatric sleeve gastrectomy patients with GERD patients was considered off-label. This landmark study demonstrated MSA to be effective for management of GERD after sleeve in the majority of patients. A larger proportion of patients in that study did have hiatal hernia repair. In that study, GERD-HRQL and the symptom of regurgitation essentially normalized at 1-year follow-up and PPI use improved. In our study, patients are followed even further, and durability of this management method is remarkable. In the bypass group all the patients did well with the MSA device.

To achieve the results seen with MSA, the authors chose a very thoughtful algorithm for choosing which patients should have this approach for GERD management after sleeve. The algorithm is in Fig. 1 [9]. Briefly patients who had BMI qualifying for weight loss surgery, abnormal sleeve morphology or esophageal dysmotility (same parameters as those without altered gastric anatomy) were managed with conversion to gastric bypass and any hiatal hernias were fixed. Manometric findings further helped define which patients had LES augmentation. If the LES pressure was reasonable (above 18 mmHg), then MSA was not used. For those with LES pressure under 18, MSA was used. Since the inception of this review more has been learned about where MSA is most helpful [18].

In this study, the explantation rate was 19%, which is higher than that seen in the literature for all comers (5%) [17]. In this study, 3 patients were explanted. None of these were among the bypass group. The average time from placement to explanation was 3–23 months with an average of 16 months. Persistent dysphagia was the most common indication for explantation (2/3) patients. Although some dysphagia is expected after MSA, most dysphagia is expected to resolve within 12 weeks of placement. Patients need to have extensive coaching on mindful eating through this period to facilitate resolution of dysphagia and keeping too much scar from forming around the beads. It is notable that all three patients had extremely low or negligible LES pressures at the time of MSA placement. In general, we have also learned that in order for MSA to work, there needs to be some baseline LES function in order for the augmentation device to work. This observation suggests that patients with negligible LES pressures (less than or equal to 5 mmHg) should not have an MSA device placed as it is unlikely to be effective. What is interesting is that at long-term follow-up, even after explantation, patients noted persistent improvement in reflux symptoms. There were no migrations or erosions in this series.

The biggest limitation within our study was our small sample size. The patients who qualified for the use of MSA as part of the management strategy is a small subset of all patients who present with reflux after weight loss surgery. However, this management algorithm does help some patients to avoid conversion to gastric bypass. Now that MSA is on label use following sleeve, hopefully larger series will accumulate in future. However, with the high explantation rate in this study, it is clear that MSA should not be used for all patients who have GERD after weight loss surgery. Another factor was that there was not 100% patient retention in our long-term follow-up with only 50% of the patients followed long term. Even with these limitations, the long-term data are promising with significant improvement in GERD-HRQL scores and symptoms. Further studies need to be performed within multicenter studies to further evaluate the long-term benefits with the use of MSA in post-bariatric surgical patients.

## Conclusion

This study suggests that the MSA device is a durable strategy in the management GERD following a prior sleeve gastrectomy or Roux-en-Y gastric bypass. Our long-term data are promising with significant improvement in GERD-HRQL scores, reduction in PPI use, and reduction in patient-reported symptoms that is durable beyond three years. The authors emphasize that patient selection is very important to achieving these outcomes. Utilizing the author’s algorithm, patients with BMI < 35, normal sleeve morphology, low pressure within the sleeve, and LES pressures < 18 mmHg, the MSA device is an effective and durable option with long-term benefits in the management of GERD following bariatric surgery.
